# Sporotrichosis during pregnancy: A retrospective study of 58 cases in a reference center from 1998 to 2023

**DOI:** 10.1371/journal.pntd.0012670

**Published:** 2024-12-20

**Authors:** Dayvison Francis Saraiva Freitas, Rodrigo Pernas Cunha, Raquel de Vasconcellos Carvalhaes de Oliveira, Priscila Marques de Macedo, Antonio Carlos Francesconi do Valle, Ana Paula Marinho Barbosa Rezende, Rosangela Vieira Eiras, André Luiz Land Curi, Erika Moreira Carvalho, Raissa Lima de Moraes, Rodrigo Almeida-Paes, Rosely Maria Zancopé-Oliveira, Maria Clara Gutierrez–Galhardo

**Affiliations:** 1 Fundação Oswaldo Cruz, Instituto Nacional de Infectologia Evandro Chagas, Laboratório de Pesquisa Clínica em Dermatologia Infecciosa, Rio de Janeiro, Brazil; 2 Fundação Oswaldo Cruz, Instituto Nacional de Infectologia Evandro Chagas, Pós-graduação em Pesquisa Clínica em Doenças Infecciosas, Rio de Janeiro, Brazil; 3 Fundação Oswaldo Cruz, Instituto Nacional de Infectologia Evandro Chagas, Laboratório de Epidemiologia Clínica, Rio de Janeiro, Brazil; 4 Fundação Oswaldo Cruz, Instituto Nacional de Infectologia Evandro Chagas, Serviço de Enfermagem, Coordenação de Atenção a Pacientes Externos, Rio de Janeiro, Brazil; 5 Fundação Oswaldo Cruz, Instituto Nacional de Infectologia Evandro Chagas, Laboratório de Pesquisa Clínica em Oftalmologia Infecciosa, Rio de Janeiro, Brazil; 6 Fundação Oswaldo Cruz, Instituto Nacional de Infectologia Evandro Chagas, Serviço Médico, Hospital Dia, Rio de Janeiro, Brazil; 7 Fundação Oswaldo Cruz, Instituto Nacional de Infectologia Evandro Chagas, Laboratório de Micologia, Rio de Janeiro, Brazil; Albert Einstein College of Medicine, UNITED STATES OF AMERICA

## Abstract

**Background:**

Pregnant women constitute a vulnerable population occasionally affected by zoonotic sporotrichosis. Treatment is challenging due to potentially teratogenic oral medications (itraconazole and saturated potassium iodide solution) or lack of clinical experience during pregnancy (terbinafine). Literature is scarce on sporotrichosis and pregnancy, mainly consisting of case reports.

**Methodology/principal findings:**

This study consists of a cohort of 58 cases of pregnant women with sporotrichosis attended in a reference center in Rio de Janeiro from 1998 to 2023. The median age was 27 years old; the majority were black (64.4%); comorbidities prior to pregnancy were reported by 20.7% (including two people living with HIV/AIDS–PLHIV) and 6.8% developed conditions that are unique to pregnancy. In 75.9% of patients, they were pregnant when they acquired sporotrichosis, with a median gestational age of 17 weeks, and 24.1% became pregnant during treatment for sporotrichosis. The lymphocutaneous form occurred in 63.8% of patients, followed by the fixed form (19%), disseminated cutaneous form (12%) and extracutaneous/disseminated forms (5.2%). Thermotherapy was indicated for all (except 2) patients and cryosurgery was performed in 22 (37.9%). Amphotericin B was indicated for a patient with external ocular sporotrichosis and for the PLHIV with osteomyelitis in the right tibia. Cure occurred in 100% of those followed (n = 44) with the remainder lost to follow-up (n = 14)

**Conclusions:**

Pregnant women with cutaneous sporotrichosis in this study recovered following physical therapies, suggesting these therapies may be effective. In cases of extracutaneous and disseminated forms, amphotericin B was indicated due to its safety profile in this population. Management of sporotrichosis during pregnancy requires a delicate assessment of the balance between maternal benefit and fetal risks.

## Author summary

Pregnant women constitute a vulnerable population occasionally affected by cats with sporotrichosis. Treatment is challenging due to oral medications that can potentially harm the baby or lack of clinical experience during pregnancy. This study consists of an analysis of 58 cases of pregnant women with sporotrichosis attended in a reference center. The median age was 27 years old and the majority were black (64.4%). The lymphocutaneous form (63.8%), fixed form (19%) and disseminated cutaneous form (12%) of sporotrichosis were treated with thermotherapy and cryosurgery (n = 22, 37.9%). For extracutaneous/disseminated forms (5.2%) (external ocular sporotrichosis and bone sporotrichosis), amphotericin B was indicated. Cure occurred in 100% of those followed (n = 44) with the remainder lost to follow-up (n = 14). For pregnant women with cutaneous sporotrichosis, the physical therapies used were effective. In cases of extracutaneous and disseminated forms, amphotericin B was indicated due to its safety profile in this population.

## Introduction

Sporotrichosis is a subacute or chronic mycosis of animals and humans, globally distributed, but most frequently found in tropical and subtropical regions. It is caused by dimorphic fungi belonging to the genus *Sporothrix*, which lives in soil and decaying organic material. The most common species of *Sporothrix* that cause sporotrichosis in humans are *Sporothrix schenckii*, *Sporothrix brasiliensis*, *Sporothrix globosa*, *Sporothrix mexicana*, and *Sporothrix luriei*. The traditional route of infection is traumatic inoculation via plant thorns and decomposing wood. Zoonotic transmission, especially from infected cats, and fungal conidial inhalation from the environment are other routes of infection [1,2].

Since the late 90’s, sporotrichosis associated with cats is a major public health problem in Rio de Janeiro, Brazil. *Sporothrix brasiliensis* is associated with this form of zoonotic transmission [3–6]. Since then, it has been reported in other Brazilian states, other countries in South America, and in Europe [6,7]. It is an emerging and the most virulent species of the genus *Sporothrix*, according to observations both in experimental animal models and in severe clinical presentations, especially in immunosuppressed hosts [8–12]. In this transmission profile, there is a predominance of women, many of them of childbearing age, who care for domestic animals [3–5]. Pregnant women are a vulnerable population occasionally affected by the zoonosis [13–16]. They are constituted by pregnant women who acquire the infection through exposure to cats with sporotrichosis; or those who are being treated for sporotrichosis and become pregnant due to failure or absence of a contraceptive method.

During pregnancy, significant systemic immunological adaptation occurs with highly dynamic cooperative interactions between the maternal and fetal immune systems. Furthermore, pregnancy continues to be one of the most vulnerable periods in terms of morbidity and mortality, for both the mother and the fetus [17]. In the case of sporotrichosis, there is no reported risk of the infection disseminating to the fetus nor is sporotrichosis worsened with pregnancy [18]. Pregnant women should not receive itraconazole due to the teratogenic potential, nor should they be treated with saturated solution potassium iodide due to its toxicity to the fetal thyroid. Terbinafine is classified by the US Food and Drug Administration as a pregnancy category B drug, meaning it is not expected to harm an unborn baby, but clinical experience during pregnancy is insufficient. Amphotericin B is recommended for severe cases of sporotrichosis that need to be pharmacologically treated during pregnancy. Local hyperthermia is indicated to treat cutaneous sporotrichosis in pregnant women and cryosurgery with liquid nitrogen spray has been used successfully in some cases [16,18–21].

Given the importance of the dynamics of sporotrichosis, with changes in its epidemiology and more serious clinical forms, its study in pregnant women is pertinent. Literature is scarce on sporotrichosis and pregnancy and mainly consists of case reports [13–16,22–24]. The goal of this study was to evaluate the clinical and epidemiological characteristics, as well as the evolution and outcomes, of pregnant women with sporotrichosis, in the largest case series reported to date worldwide, from a reference center in a region of zoonotic transmission in Rio de Janeiro.

## Methods

### Ethics statement

This study was approved by the Fundação Oswaldo Cruz (Fiocruz), Instituto Nacional de Infectologia Evandro Chagas (INI) Ethics Committee (#15554419.0.0000.5262), in accordance with the Brazilian ethical standards. The accessible patients signed an informed consent form, while the others were exempted by the Ethics Committee. The researchers signed a Term of Commitment and Responsibility, assuring confidentiality of the data.

### Study location

This study was carried out at Fiocruz, INI, linked to the national Ministry of Health, which has been a primary reference center for sporotrichosis cases in the hyperendemic area of Rio de Janeiro, Brazil.

### Patients and study design

We conducted an observational and retrospective study on a cohort of patients with culture-proven sporotrichosis and evidence of concomitant pregnancy, from 1998 to 2023 [3].

For that, a search was carried out at the Institute’s Information and Communication Technology Service and a cross-check with the databases of the Clinical Research in Infectious Dermatology Laboratory to retrieve the records of patients who were pregnant at some point during treatment for sporotrichosis in the proposed period. The terms “gestation”, “pregnant woman”, “pregnancy” and “pregnant” were used as keywords to search the electronic medical records. Patients who did not have mycological confirmation of sporotrichosis were excluded from this study.

### Patient management

The initial follow-up of patients with sporotrichosis at Fiocruz, INI, followed a protocol that involved clinical evaluation, mycological examination (culture) and blood tests (blood count, biochemistry and liver function). Other tests were performed according to the need to investigate and monitor extracutaneous forms of sporotrichosis. In the anamnesis of female patients of childbearing age (≥ 14 years and ≤ 45 years), if a menstrual delay or suspicion of pregnancy was reported, a urine pregnancy test was requested before starting pharmacological treatment.

In the case of pregnant women with sporotrichosis, they were advised to treat sporotrichosis with home thermotherapy with local heat, applying three daily cycles, 20 minutes each, at a temperature of approximately 42–43°C, using a compress with a thermal bag. Cryosurgery was performed during medical consultation, in ulcerated lesions (nodular-ulcerated and vegetative ulcers), verrucous and nodular (exophytic) lesions. Each lesion was subjected to two cycles of freezing and thawing using the intermittent spray technique. The cycles varied from 10 to 30 seconds, with a freezing halo of 3 to 5 mm. Analgesia (dipyrone 500 mg or paracetamol 500 mg, both given orally) could be administered pre-procedure, when necessary. Cryosurgery sessions were performed monthly, while the lesions to be treated persisted [16,25]. In cases of extracutaneous and disseminated forms, amphotericin B lipid complex (3–5 mg/kg/day) was indicated until clinical cure.

Monthly assessments were carried out until the lesions were healed. The patient was then reevaluated between one and three months later, at which point she was definitively discharged. Recurrence (or relapse) was considered to have taken place if the lesion returned after healing.

The nursing team also participated in caring for pregnant women, providing clarification and guidance on how to care for skin lesions. Follow-up consultations were scheduled with greater flexibility and individualization with the aim of closely monitoring the evolution of the infection in these patients.

After delivery, if the patient had active sporotrichosis lesions and was not breastfeeding or did not want to breastfeed, options for using oral medications were discussed. Itraconazole (100 to 200 mg/day) was the first option for cutaneous forms, and terbinafine (250 to 500 mg/day), in case of contraindication [21].

### Analysis of collected data

Clinical, epidemiological, and laboratory data were collected from patients’ medical records. These data were anonymized and re-identified to protect the patients’ privacy and confidentiality. The variables were stored in a database (Microsoft Excel version 2013) and analyzed with the survival package of the open-access program R version 4.4.0.

Due to the great ethnic diversity in Brazil, we defined the skin color/race variable as corresponding to white or black (brown/mixed-black and black). Ethnicity/color was established by the administrative staff at the time of registration in the institute until the year 2005; subsequently, this information was self-reported by the patients according to their ancestry. Gestational age was established based on the patient’s self-reporting or based on parameters determined by the uterus ultrasound performed. The outcome of sporotrichosis was defined as cure (that is, re-epithelialization of the lesions with absence of crusts, exudate, erythema and infiltration), no cure and loss of follow-up. The outcome of pregnancy was defined as favorable (normal, after the 37th week of gestation); and unfavorable, miscarriage or premature birth (before completing 37 weeks of pregnancy). The outcome of the newborn was defined as favorable (healthy newborn) and unfavorable (stillborn or low birth weight or newborn with alteration in morphogenesis).

Frequencies, percentages, and summary measures such as median and interquartile range (IQR) were calculated. In the calculation of frequencies in terms of percentages, valid cases were considered, and missing data were excluded. Statistical analysis was conducted on categories of quantitative variables. Comparisons were made between those patients exposed to contraindicated medications for the treatment of sporotrichosis in pregnant women (itraconazole and saturated solution potassium iodide) and those not exposed, with pregnancy and newborn outcomes. Specifically, the Mann–Whitney test was used for quantitative data, whereas the Fisher exact test was used for the qualitative variables. All hypothesis tests were performed considering a significance level of 5%.

## Results

**Fig 1:**
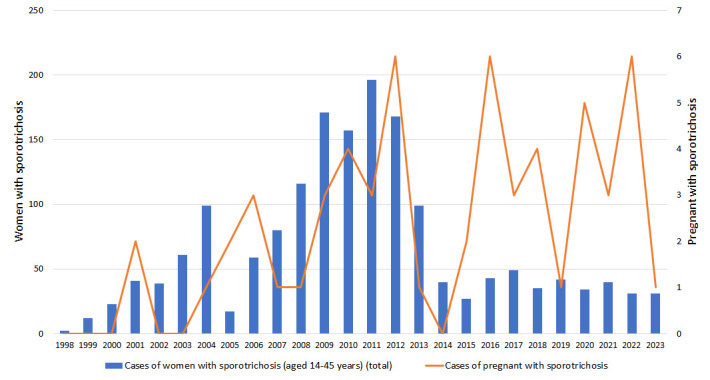
Annual frequency of the total number of cases of women of childbearing age (≥ 14 years and ≤ 45 years) with sporotrichosis and cases involving pregnant women attended at Fiocruz, INI, from 1998 to 2023.

From 1998 to 2023, 3,727 women were diagnosed with sporotrichosis at Fiocruz, INI. Of these, 94 were pregnant and 36 were excluded from this study because they did not have mycological confirmation of sporotrichosis. The first pregnant woman with culture-proven sporotrichosis was diagnosed in 2001. The annual frequency of the total number of cases of women of childbearing age (≥ 14 years and ≤ 45 years) with sporotrichosis and the cases involving pregnant women can be seen in Fig 1. Fifty-eight pregnant women with sporotrichosis were included, representing 3.4% of 1,712 women with this mycosis of childbearing age attended in our institution during this period.

### 3.1. Socio-Demographic and Epidemiological Characteristics

The age of the patients ranged from 15 to 41 years, with a median age of 27 (IQR = 23–33.7) years old.

Of the 45 patients with available skin color/race data, 64.4% (n = 29) were black and 35.6% (n = 16) were white. Regarding the level of education, 20.4% (n = 11) had up to 9 years of education, 70.4% (n = 38) 12 years and 9.2% (n = 5) 16 or more years of study. As for their occupations (n = 54), the most common were housewives (33.3%, n = 18) and students (14.8%, n = 8). Other activities were grouped due to lower proportions of cases: commercial activities (14.8%, n = 8), nurse/nursing assistant/caregiver (9.2%, n = 5), manicurist/hairdresser/beautician (7.4%, n = 4), teacher (3.7%, n = 2) and miscellaneous (13%, n = 7, laundry assistants, bank clerks, seamstresses, public servants, military servants and telephone operators). Two patients (3.7%) were unemployed.

Most of the 58 cases (n = 56, 96.5%) originated in high-density areas of zoonotic sporotrichosis in Rio de Janeiro and a group of cities in the same metropolitan region. Regarding possible sources of infection, 52 patients reported contact with cats and for six patients this information was not available. Scratching or biting were reported by 31 (59.6%) patients, whereas 21 (40.4%) patients denied any trauma that preceded the lesions.

### 3.2 Temporal relation between pregnancy and sporotrichosis

Forty-four (75.9%) patients were pregnant when they acquired sporotrichosis, with a median gestational age of 17 (range: 4–40) weeks. Fourteen patients (24.1%) became pregnant during sporotrichosis treatment. Nine were from the Fiocruz, INI sporotrichosis cohort and five were being treated in other external units. Two patients had twin pregnancies.

### 3.3. Comorbidities

Among the 58 patients, 12 (20.7%) reported comorbidities prior to pregnancy such as high blood pressure (n = 4), HIV infection (n = 2), psychiatric disorders (n = 2), diabetes (n = 1), high blood pressure and diabetes (n = 1), asthma (n = 1) and arthrosis (n = 1).

### 3.4. Clinical forms of sporotrichosis

**Fig 2:**
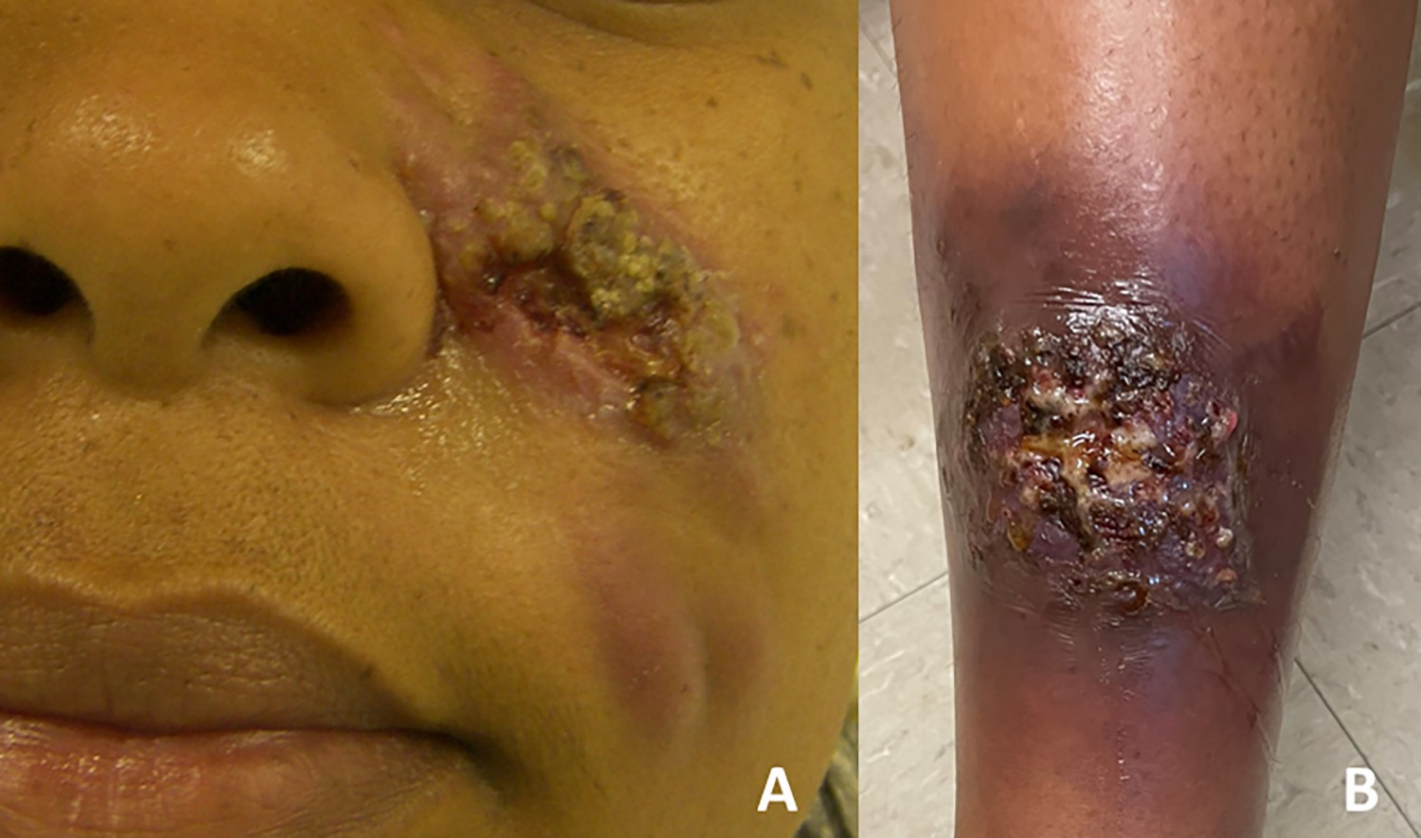
Pregnant women with lymphocutaneous sporotrichosis. A) Case 01 with nodular-ulcerated lesion and nodules/abscesses on the left hemiface. B) Case 48 with detail of the main lesion, with a very friable and inflammatory ulcerated plaque on the left leg.

**Fig 3:**
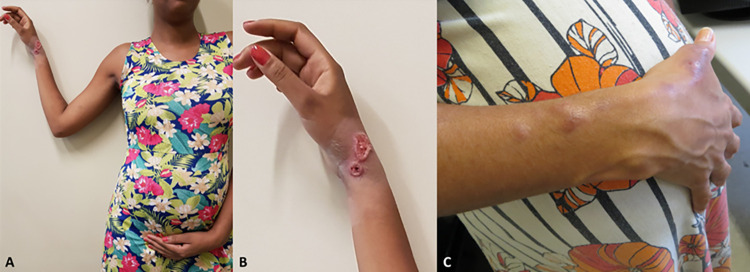
Pregnant women with lymphocutaneous sporotrichosis in the right upper limb. A) Case 14, who underwent three cryosurgery sessions (three months of treatment) and was cured; B) Her lesions in detail. C) Case 21, who was treated with thermotherapy and fine needle aspiration of nodules/abscesses.

Most of the 58 patients (63.8%, n = 37) presented the lymphocutaneous form (Figs 2 and 3), followed by fixed cutaneous form (19%, n = 11), disseminated cutaneous form (12%, n = 7) and extracutaneous/disseminated form (5.2%, n = 3). Case 8, a person living with HIV (PLHIV) with the fixed form of sporotrichosis, had a T CD4^+^ count of 309 cells/mm^3^ and a viral load of 4,512 copies/mL.

**Fig 4:**
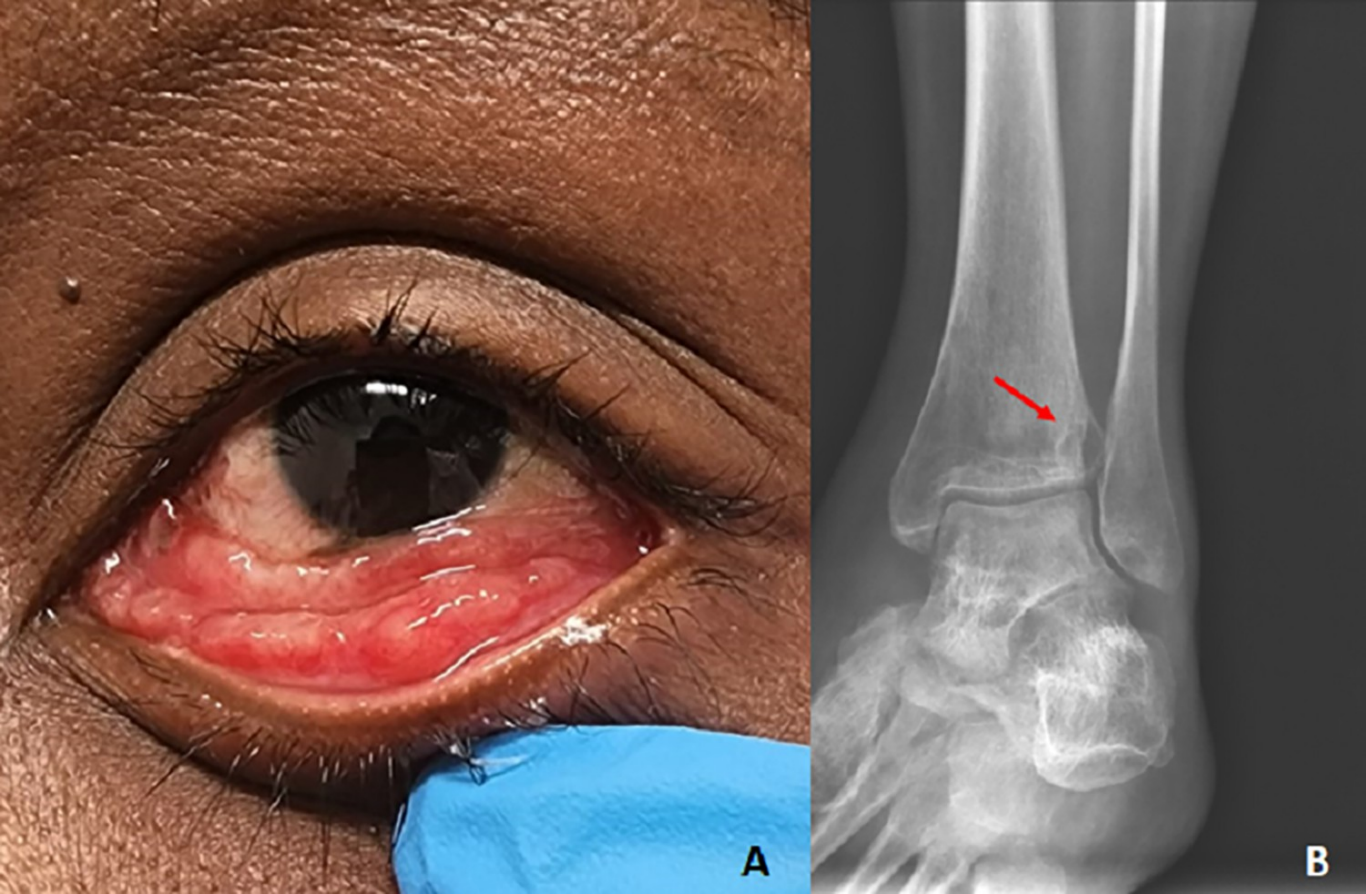
Pregnant women with extracutaneous/disseminated sporotrichosis. A) Case 52 with ocular sporotrichosis, presenting exuberant granulomatous conjunctivitis affecting the tarsal and bulbar conjunctiva of the left eye. B) Case 15 was a patient living with HIV with bone sporotrichosis lesion. Radiograph of the left foot and ankle showed a small lytic lesion in the distal tibia (red arrow), next to the lateral malleolus. Credit: Service of Image of Fiocruz, INI.

In the group of patients with extracutaneous/disseminated sporotrichosis, one had granulomatous conjunctivitis in the left eye (Fig 4A); another one had lymphocutaneous and nasal sporotrichosis; and the third (case 15) was a PLHIV. She had been treated for 14 months using itraconazole with regression of cutaneous and oropharyngeal mucosa lesions and osteomyelitis in the right tibia. In this occasion, her T CD4^+^ count was 453 cells/mm^3^ with an undetectable viral load. However, she had a small focus of osteomyelitis in the left tibia (Fig 4B), when she became pregnant. Two patients with the lymphocutaneous form and one with the fixed cutaneous form presented the hypersensitivity reactions erythema nodosum, arthralgias and erythema multiforme, respectively.

In relation to the topographic distribution in the patients, the lesions were on the upper limbs (70.7%, n = 41), followed by the lower limbs (20.7%, n = 12), trunk (13.8%, n = 8) and head (12%, n = 7). Seven (13.8%) patients had lesions on more than one body segment.

### 3.5. Treatment and outcome of sporotrichosis

**Fig 5:**
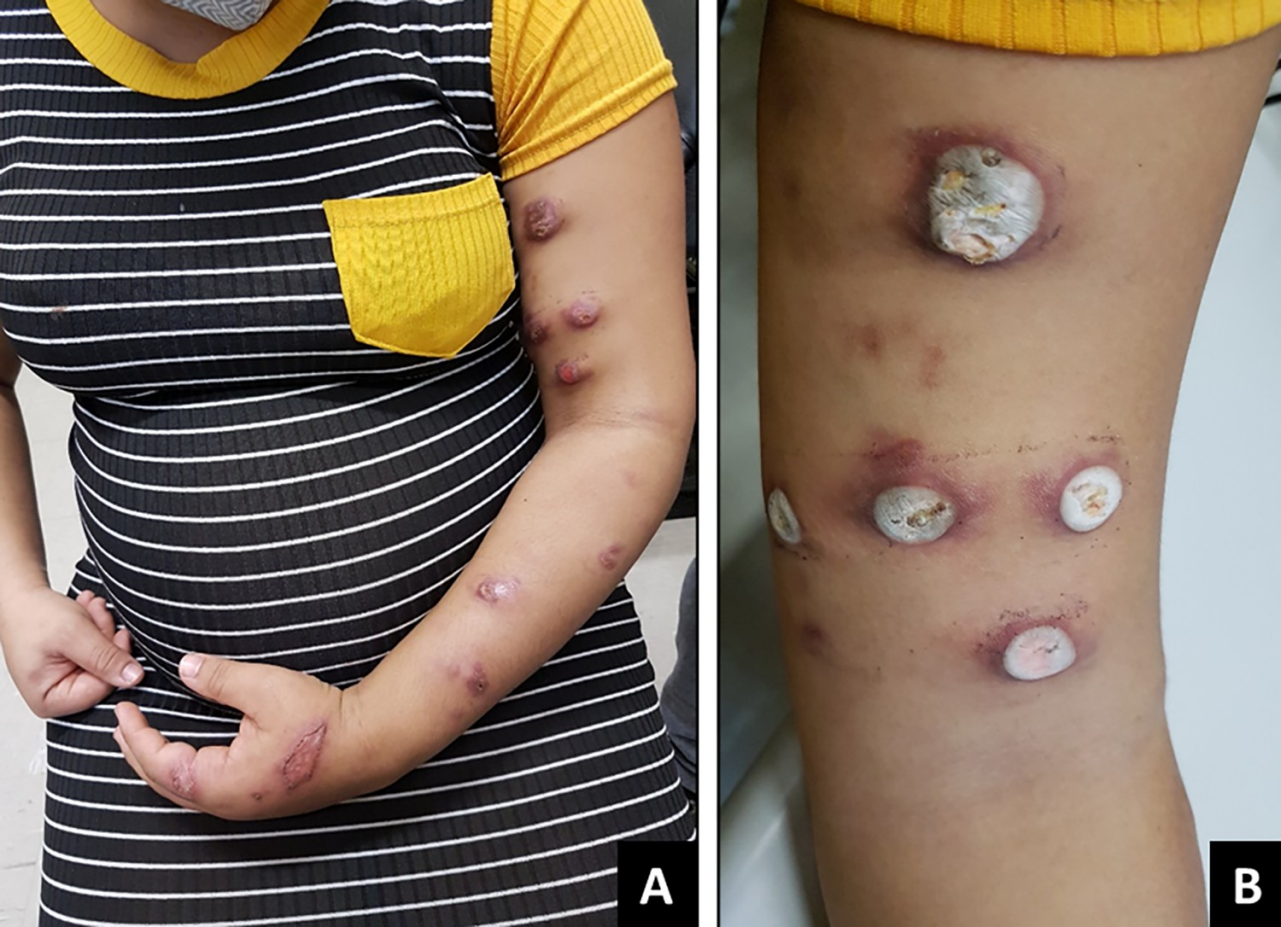
Pregnant woman with lymphocutaneous sporotrichosis in the left upper limb (case 40). A) Ascending nodular-ulcerated lesions in a healing process. B) Some lesions immediately after cryosurgery, showing freezing.

Thermotherapy was indicated for all patients with cutaneous lesions (n = 56); cryosurgery was performed in 22 (37.9%) patients (Fig 5), with a median of 2 (range: 1–7) sessions; and nodule/abscess aspiration in 4 (6.9%) patients.

**Fig 6:**
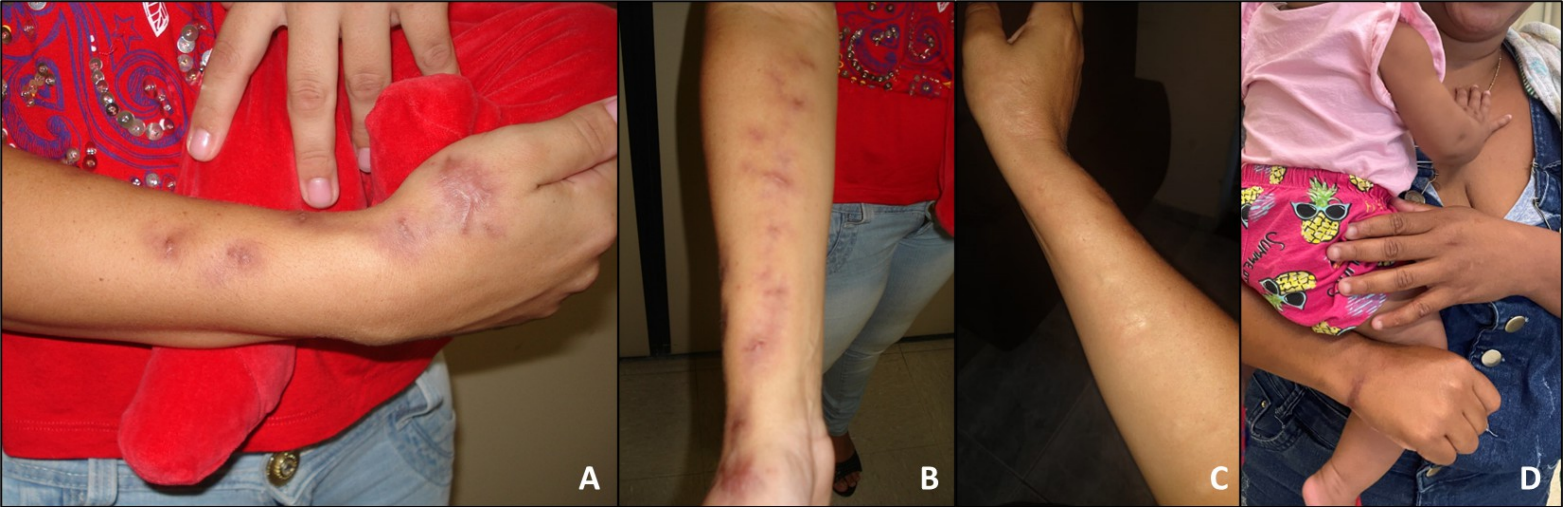
Mothers with lymphocutaneous sporotrichosis scars on the right upper limb. A, B) Case 16 with recent pink scar in the postpartum period. C) Scar 10 years later, flatter and normochromic. D) Case 54 with a hyperchromic scar on the right wrist taking her healthy 7-month-old daughter to the discharge consultation. Treatment lasted 35 days, with local heat at home and three outpatient cryosurgery sessions.

Eighteen (31.0%) were exposed to sporotrichosis medications contraindicated for pregnant women (itraconazole and saturated solution potassium iodide). Fifteen (83.3%) were exposed because the pregnancy status was unknown and three (16.7%) due to inadequate prescription, out of our institution. The majority (72.2%, n = 13) of them were in the first trimester of pregnancy. All these patients had their medication suspended once both conditions (sporotrichosis and pregnancy) were established at Fiocruz, INI. As case 15 was a PLHIV with bone sporotrichosis, she was referred to another hospital that offered high-risk pregnancy and infectious disease care, for treatment with amphotericin B. Case 52, with conjunctival sporotrichosis, was prescribed lipid complex amphotericin B (200 mg *per* infusion, 14 infusions over three and a half months) by the ophthalmology team. At the end of pregnancy, nine patients with persistent sporotrichosis lesions who wanted to optimize treatment were prescribed itraconazole. Seven of them were given it as a decision made between doctor and patient, because they would not breastfeed, and two because they suffered a miscarriage. All the 44 patients who completed follow-up were cured, with a median of 122.5 (range: 35–458) days and a median of 6.5 (range 2–30) consultations performed (Fig 6).

**Fig 7:**
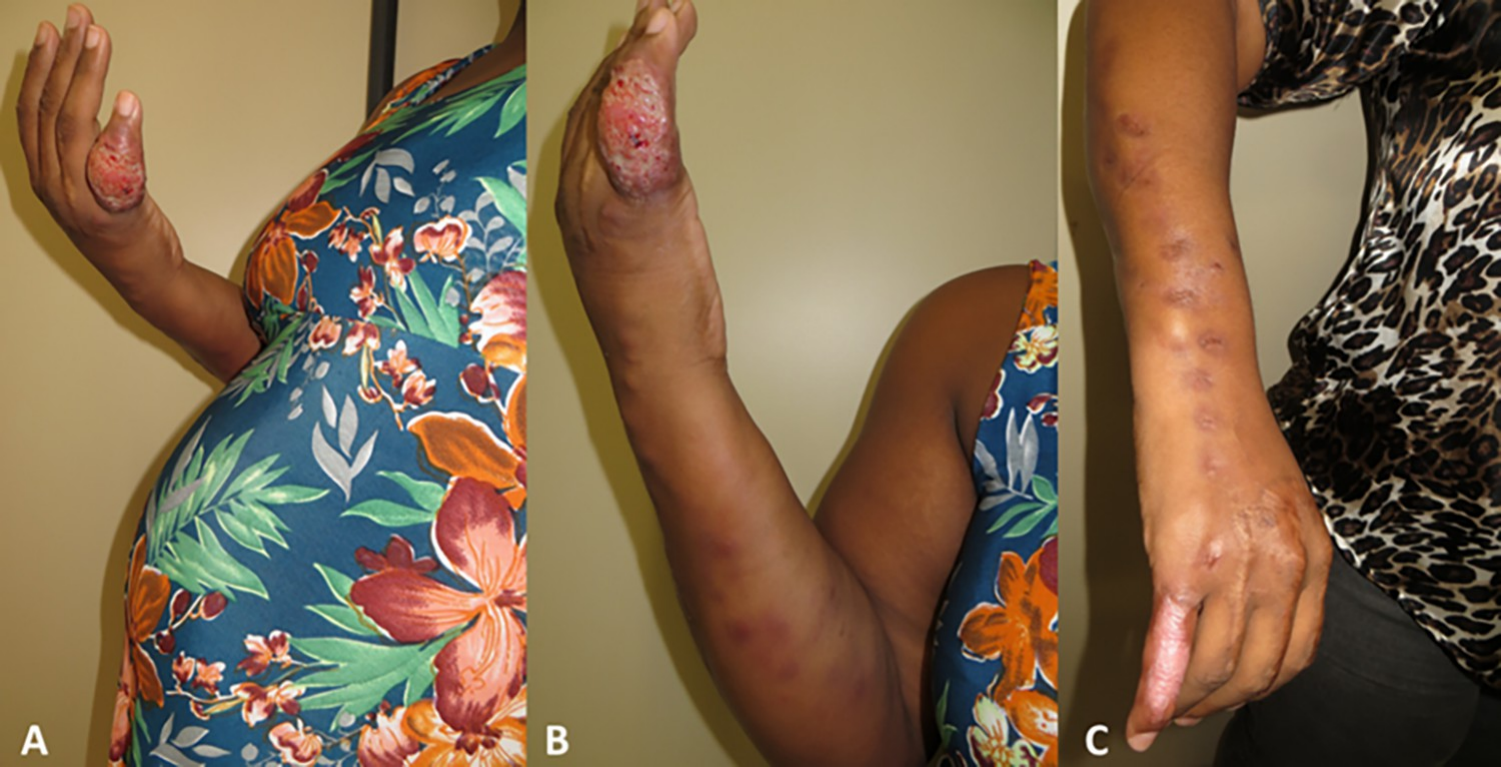
Case 10 with lymphocutaneous sporotrichosis on the right upper limb, where she had burn scars. She was treated during pregnancy with cryosurgery and thermotherapy. After delivery, she did not breastfeed and used itraconazole 200 mg/day for six months until healing. The disease relapsed one month later and itraconazole was reintroduced at the same dosage, with cure after additional two months. A, B) Lesions at the beginning of treatment, during pregnancy. C) Postpartum, already cured.

Three patients who had used itraconazole after the end of pregnancy, relapsed (Cases 10, 20 and 53). Itraconazole was restarted with the same dosage (100 or 200 mg/day), and they were subsequently cured (Fig 7).

Fourteen (24.1%) were lost to follow-up. Two were PLHIV who were referred for treatment in other health units; the remainder had no comorbidities. The median days of treatment for these patients was 90 (range 1 to 350); and the median of consultations was 3 (range 1–9). Six patients (42.8%, n = 14) had almost complete regression of the lesions at the last consultation before the loss to follow-up. When we evaluate the age of the patients who were lost to follow-up, they were younger (median age 23, range: 15–40 years old) compared to those who completed treatment (median age 27.5, range: 16–41 years old) *(p = 0*.*0372)*.

Table 1 presents a compilation of the main features observed in the patients with sporotrichosis included in this study.

**Table 1:** Clinical, demographic data and outcome of pregnant patients with sporotrichosis attended at Fiocruz, INI, from 2001 to 2023.

Case	Age (years)	Pregnancy (weeks)	Mother disease	Clinical form	Treatment	Outcome of Sporotrichosis
**1**	34	DT^a^*	-	LC	heat / ITZ^b^ after	cure
**2**	16	13	-	LC	heat	lost
**3**	25	34	toxoplasmosis	LC	heat	cure
**4**	33	13	-	FC	heat	lost
**5**	35	17	-	LC + arthralgias	heat	cure
**6**	22	DT	psychiatric disorder	LC	heat	cure
**7**	32	28	-	DC	heat	cure
**8**	21	21	HIV infection	FC	heat + cryo^c^	lost
**9**	20	38		FC	heat	cure
**10**	38	36	asthma	LC	heat + cryo / ITZ after	cure^f^
**11**	36	DT	HBP^d^	LC	heat	cure
**12**	26	DT	preeclampsia	LC	heat + cryo / ITZ after	cure
**13**	33	21	-	LC	heat	cure
**14**	29	13	-	LC	heat + cryo	cure
**15**	27	DT	HIV infection	Disseminated	Amphotericin B	lost
**16**	27	17	-	LC	heat + FNA^e^	cure
**17**	26	21	-	FC + EM	heat	cure
**18**	18	21	-	FC	heat + cryo	lost
**19**	25	21	-	LC	heat	cure
**20**	41	DT	HBP	LC	heat / ITZ after	cure^f^
**21**	35	16	-	LC	heat + FNA	cure
**22**	24	17	-	DC	heat	cure
**23**	17	36	-	LC	heat + cryo	cure
**24**	38	DT	diabetes	LC	heat + cryo	cure
**25**	35	17	-	LC	heat	cure
**26**	23	28	-	LC	heat	lost
**27**	32	12	HBP + gestational diabetes	DC	heat + FNA + curettage	cure
**28**	27	DT	-	LC	heat	cure
**29**	22	17	-	LC	heat + cryo	cure
**30**	23	30	-	LC	heat + cryo / ITZ after	cure
**31**	40	DT	psychiatric disorder	LC	heat	cure
**32**	15	22	-	LC	heat + cryo + FNA	lost
**33**	28	DT	-	LC	heat	cure
**34**	23	DT	-	DC	heat	lost
**35**	30	DT	-	LC	heat + cryo	cure
**36**	32	14	-	LC	heat + cryo	cure
**37**	16	12	-	LC	heat	cure
**38**	40	4	arthrosis	FC	heat / ITZ after	lost
**39**	23	29	-	DC	heat	cure
**40**	26	24	-	LC	heat + cryo	cure
**41**	21	15	-	FC	heat + cryo	cure
**42**	27	DT	-	LC + nasal	heat	cure
**43**	18	8	-	LC + EN	heat	lost
**44**	26	DT	-	LC	heat	cure
**45**	25	28	-	FC	heat / ITZ after	cure
**46**	38	4	-	LC	heat	cure
**47**	28	12	-	LC	heat	lost
**48**	27	22	-	LC	heat + cryo	cure
**49**	27	17	gestational diabetes	FC	heat + cryo	cure
**50**	27	14	-	DC	heat + cryo	cure
**51**	20	11	-	LC	heat	lost
**52**	35	8	HBP + diabetes	external eye disease	Amphotericin B	cure
**53**	34	DT	-	LC	heat + cryo / ITZ after	cure^f^
**54**	35	28	HBP	LC	heart + cryo	cure
**55**	27	38	-	FC	heat	lost
**56**	35	12	-	LC	heat + cryo	lost
**57**	32	40	-	FC	heart + cryo / ITZ after	cure
**58**	32	12	-	DC	heat + cryo	cure

^a^DT = patient became pregnant during sporotrichosis treatment

^b^ITZ = itraconazole

^c^cryo = cryosurgery

^d^HBP = high blood pressure

^e^FNA = fine needle aspiration

^f^Cases with relapse and cure after restart of ITZ. LC = lymphocutaneous. FC = fixed cutaneous. DC = disseminated cutaneous. EN = erythema nodosum. EM = erythema multiforme.

*DT patients exposed to sporotrichosis medications contraindicated for pregnant women because the pregnancy status was unknown. Cases 7, 10 and 17 were exposed to sporotrichosis medications contraindicated for pregnant women due to inadequate prescription, out of our institution.

### 3.6 Pregnancy and newborn outcomes

Four patients (6.8%) developed conditions during the pregnancy such as gestational diabetes (n = 1), gestational diabetes and high blood pressure (n = 1), preeclampsia (n = 1) and toxoplasmosis (n = 1).

The pregnancy outcome of 36 patients was analyzed (missing value = 22). Of these, 29 (80.6%) patients reported no complications (favorable outcome) and 7 (19.4%) had complications (unfavorable outcome). As for the outcomes of the newborns (n = 35), 28 (80.0%) were healthy (favorable outcome) and 7 (20.0%) had an unfavorable outcome (missing value = 22, miscarriage = 3). Table 2 presents clinical and demographic data of ten pregnant women with sporotrichosis with unfavorable outcomes (pregnancy and newborn).

**Table 2:** Clinical and demographic data of pregnant patients with sporotrichosis with unfavorable outcome (pregnancy and newborn) attended at Fiocruz, INI, from 2001 to 2023.

Case	Age (years)	Pregnancy (weeks)	Mother disease	Medication used during pregnancy	Outcome	
					Pregnancy*	Newborn**
**11**	36	5	HBP^a^	ITZ^b^ + SSKI^c^	premature birth	low birth weight
**12**	26	26	preeclampsia	ITZ	premature birth (eclampsia)	stillborn
**13**	33	21	-	-	premature birth (twin pregnancy)	low birth weight and stillborn
**20**	41	6	HBP	ITZ	miscarriage	-
**24**	38	DT^d^	diabetes	ITZ	miscarriage	-
**29**	22	17	-	-	normal	stillborn (congenital toxoplasmosis)
**31**	40	DT	psychiatric disorder	ITZ	normal	newborn with intraventricular communication
**35**	30	DT	-	ITZ	premature birth	healthy
**38**	40	4	arthrosis	-	miscarriage	-
**46**	38	4	-	-	normal	stillborn

^a^HBP = high blood pressure

^b^ ITZ = itraconazole

^c^SSKI = saturated solution potassium iodide

^d^DT = patient became pregnant during sporotrichosis treatment.

### 3.7. Outcomes of patients exposed and not exposed to contraindicated medications for the treatment of sporotrichosis

**Fig 8:**
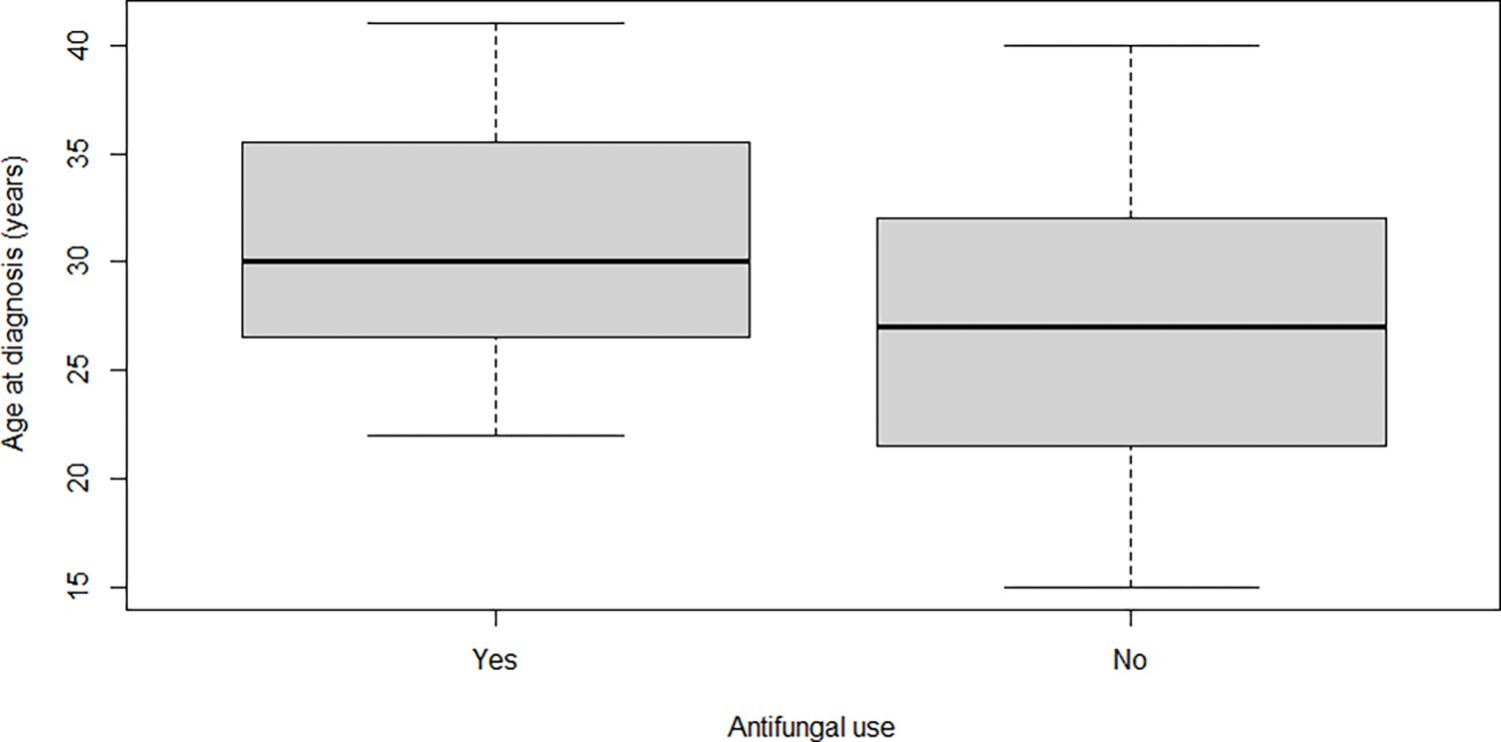
Boxplot of age of pregnant women with sporotrichosis separated by who were (or not) exposed to sporotrichosis medications that are contraindicated for pregnant women, at Fiocruz, INI, from 2001 to 2023. Patients exposed were older, with a median of 30 (IQR: 26.5–35.5, range 22–41) years old, than those who were not exposed (median of 27, IQR: 21.5–32, range: 15–40) (*p < 0*.*019*).

**Fig 9:**
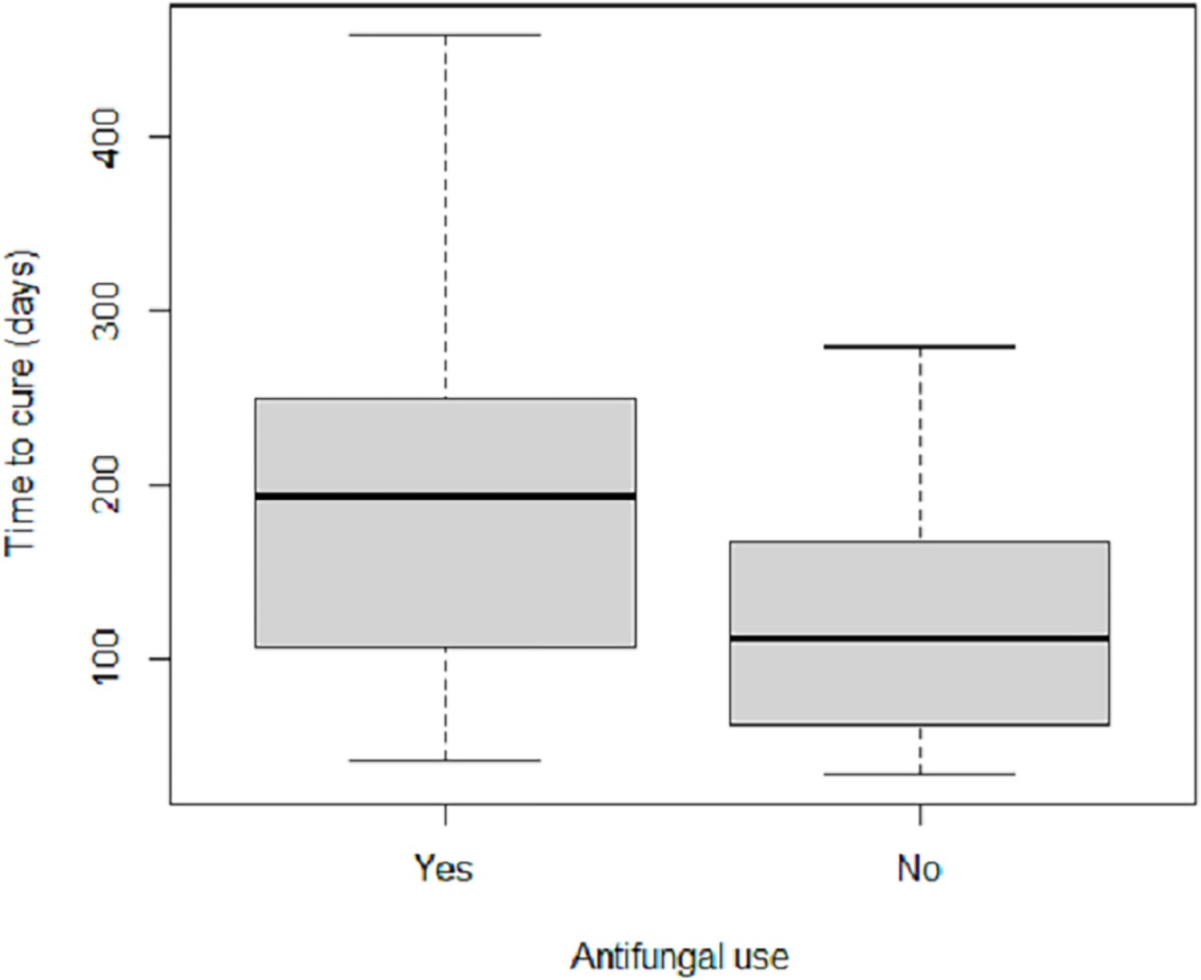
Boxplot of time to cure (days) of pregnant women with sporotrichosis separated by who were (or not) exposed to sporotrichosis medications that are contraindicated for pregnant women, at Fiocruz, INI, from 2001 to 2023. Patients exposed took longer to cure, with a median of 194 (IQR: 108–249) days *versus* 113 (IQR: 64.5–164.5) days *(p < 0*.*031)*.

Women who were exposed to sporotrichosis medications that are contraindicated for pregnant women were older *(p < 0*.*019)* (Fig 8) and they took longer to cure *(p < 0*.*031)* (Fig 9) than those who were not exposed.

When comparing the pregnancy and newborn outcomes of patients exposed and not exposed to contraindicated medications for the treatment of sporotrichosis during pregnancy, we found no statistical differences between the groups (p = 0.084 and p = 0.670, respectively). No other variable was associated with the outcome, except age at diagnosis, with a higher proportion of unfavorable pregnancy outcome among older women (*p = 0*.*014*) (S1 and S2 Tables).

## Discussion

The year 2023 was the silver anniversary of zoonotic sporotrichosis in Rio de Janeiro. Although it is a hyperendemic condition that is yet to be brought under control, there have been advances in combating it, with educational measures for transmission and prevention among the population, structuring of human care flows involving the basic network and reference centers and measures to control the disease in animals [26]. In 2013, sporotrichosis became a notifiable disease in the state and government authorities introduced public health measures to curb this mycosis in Rio de Janeiro. As a result, Fiocruz, INI began to receive referenced cases, such as PLHIV, patients with hypersensitivity reactions, and pregnancy. A relative increase in numbers of pregnant women with sporotrichosis since this period can be seen, culminating in 2022 when 19.4% of the cases of women of childbearing age receiving treatment at Fiocruz, INI were pregnant.

In this study, there was a predominance of black pregnant women, which contrasts with the dominant profile of non-pregnant white women in this form of transmission, who were not engaged in any professional activity [3,4,9]. In Brazil there is a historical socioeconomic discrepancy that keeps the black population in a situation of greater economic vulnerability and subsequently with greater difficulty in accessing referral health services [27]. Black women also occupy a more vulnerable position because they experience triple discrimination–gender, race/skin color and social class. They start prenatal care later, undergo fewer consultations and exams and receive less guidance regarding care during pregnancy and childbirth [28]. The majority of patients were infected during pregnancy, which may suggest a lack of knowledge in preventing endemic zoonoses, in this case awareness of the need to avoid contact with potentially sick or sick cats. Prenatal care is the time to strengthen such measures. For women in childbearing age undergoing treatment for sporotrichosis, it is important to reinforce the use of contraceptive methods at each consultation.

Although *S*. *brasiliensis* is considered the most virulent species and pregnant women could have a more severe evolution, all patients, except one, presented clinical forms that were benign and some typical of zoonosis sporotrichosis, such as disseminated cutaneous/extracutaneous forms (without immunosuppression) and manifestations of hypersensitivity [20,29–31]. This is different from e.g., coccidioidomycosis in which pregnancy is a risk factor for the spread of the infection and associated with high maternal and fetal mortality [32,33].

In this cohort of cases of pregnant women with sporotrichosis, all (except two) had cutaneous lesions and were treated with the physical therapies. Since 1950, thermotherapy has been reported as a successful topical method for treating cutaneous sporotrichosis. Its exact therapeutic mechanism remains obscure. In *vitro* studies showed growth inhibition of *Sporothrix* in temperatures above 38.5°C [34,35]. In a mouse model, hyperthermia was significantly more effective in eliminating skin lesions caused by *S*. *globosa* compared to the control group [36]. Thermotherapy requires a faithful application with a degree of caution to avoid skin burns. Cryosurgery has been used to treat sporotrichosis in our institution since 2001 with success, including in pregnant women. Cryosurgery is not only an ablative technique, but also promotes a local immunological response [25]. Its advantages include its relatively low cost, ease of use for professionals who have mastered the technique and a low rate of complications, generally mild and transient side effects. It is contraindicated for patients sensitive to cold (cold urticaria, cryoglobulinemia or cryofibrinogenemia) and should be avoided in extensive lesions or flexor surfaces due to the risk of fibrosis [16,25]. Fine needle aspiration of skin nodules/abscesses can be diagnostic and therapeutic, promoting their emptying (and subsequently reducing the fungal load), relieving local discomfort and pain, and accelerating healing, as done here. Amphotericin B is considered as the safest antifungal drug in pregnancy. In this study, it was indicated for chronic bone sporotrichosis which is very common in PLHIV and for a patient with primary ocular sporotrichosis due to the risk of her developing symblepharon [18,21,29,30].

All patients who completed follow-up progressed to cure, with a median time approximately one month longer than in the general population, with the same epidemiological and clinical characteristics [3]. The patients who were lost to follow-up were younger, had no comorbidities, and the majority had practically healed lesions at the last consultation. It can be assumed they were cured and chose not to attend further consultations. However, adherence measures must be instituted in this subgroup of patients.

Pregnancy is a transitory phase in a woman’s life and sporotrichosis treatment with oral medications can be postponed, if necessary, after childbirth, as occurred in this study, since the majority of sporotrichosis cases, especially those only presenting cutaneous involvement are not life-threatening. In this context, pregnant women cannot be breastfeeding, as the medications should not be administered during lactation [14,18].

Interestingly, women who were exposed to sporotrichosis medications that are contraindicated for pregnant women were older and took longer to cure when compared with those who did not. The presence of an antifungal did not seem to impact the speed of cure or result in unfavorable outcomes to the pregnancy and/or to the newborn. Without going deeper into the topic, which is beyond the scope of the study, the majority had factors that could have interfered with an unfavorable outcome such as age over 40 years, chronic metabolic/degenerative diseases and one had untreated toxoplasmosis during pregnancy [37].

Pregnancy presents an ethical dilemma in the conduct of clinical trials, so safety data is based on limited case reports, animal studies, reviews of population and hospital records, and expert opinions. Published epidemiologic studies of women exposed in the first trimester of pregnancy to itraconazole have reported no risk of major birth defects overall and inconclusive findings on the risk of miscarriage [39,40]. A meta-analysis to assess the risk of fetal malformations found no significant association but concluded that eye defects in the itraconazole-exposed population should be investigated cautiously [41]. The Danish nationwide cohort study spanning 20 years showed that oral or topical terbinafine use in pregnancy does not appear to be associated with an increased risk of major malformations, spontaneous abortion or any unfavorable pregnancy outcome [42,43]. One of the limitations of the study, considered by the authors themselves, was in the methodology. The use of terbinafine was recorded based on filled prescriptions. If pregnant women did not adhere to the medication dispensed, the association with the outcome of exposed pregnancies would be biased towards unexposed pregnancies. Another point was that these findings should be confirmed in other independent populations. We would like to highlight that, in the Danish study, exposure to terbinafine was evaluated only as a dichotomous variable (yes or no) and exposure times were not evaluated. Overall, the decision to prescribe an oral antifungal to a pregnant patient must take into account safety data that also assess the duration of treatment. In the case of cutaneous sporotrichosis, it corresponds to a period of 12 weeks, which is one trimester of pregnancy.

Finally, pregnant women with sporotrichosis need to be welcomed by the health system. Welcoming is a guideline of the national Humanization Policy and must be part of all health service meetings [44]. The number of consultations carried out (median 6.5) can be an indicator of this welcome, as we monitor the evolution of the situation more closely. Welcoming pregnant women is an ethical stance that involves listening to the user’s complaints, recognizing their role in the health and illness process and taking responsibility for activating knowledge sharing networks.

The design of this study is retrospective and subject to data loss and bias. Other limitations are related to the institutional structure. INI, Fiocruz is dedicated to infectious diseases without obstetric/prenatal and pediatric/neonatal monitoring. Therefore, for this study it was difficult to follow-up obstetric and pediatric outcomes. This was seen in a higher loss of follow-up in the sporotrichosis outcome in this group of pregnant women compared to the general cases published by our group up to now [3,5].

The management of sporotrichosis in pregnancy requires a delicate assessment of the balance between maternal benefit and fetal risks [38]. If the clinical forms are cutaneous, the ideal strategy is to adopt a conservative treatment with thermotherapy alone or associated with cryosurgery (if indicated), until the time of delivery, because spontaneous resolution is rare [13]. After that, the clinical progression can be reevaluated. Amphotericin B is indicated in extracutaneous and disseminated cases due to its safety profile in this population. Pregnant women with sporotrichosis need to be welcomed by the health system. They should be assured that they will be monitored and treated with effective therapies, and that research to date has shown that sporotrichosis does not pose risks to the baby, which is generally the mother’s greatest concern!

## Supporting information

S1 Table Analysis of demographic/clinical variables and pregnancy outcomes of patients with sporotrichosis treated at Fiocruz, INI, from 2001 to 2023. (DOCX)

S2 Table Analysis of demographic/clinical variables and neonatal outcomes of pregnant women with sporotrichosis treated at Fiocruz, INI, from 2001 to 2023. (DOCX)
