# Supplementary material for: Sporotrichosis during pregnancy: A retrospective study of 58 cases in a reference center from 1998 to 2023
Source: PLoS Negl Trop Dis. 2024 Dec 20;18(12):e0012670. doi: 10.1371/journal.pntd.0012670 (PMC11661573; doi:10.1371/journal.pntd.0012670)
Supplement: S1 Table — (DOCX) [file pntd.0012670.s001.docx]

**S1 Table.** Analysis of demographic/clinical variables and pregnancy outcomes of patients with sporotrichosis treated at Fiocruz, INI, from 2001 to 2023.

| Variable | Pregnancy outcome | | 95% CI ^b^ | Pregnancy outcome | | 95% CI ^b^ | p-value |
| --- | --- | --- | --- | --- | --- | --- | --- |
|  | **Favorable**  **N = 29 (81%)** | |  | **Unfavorable**  **N = 7 (19%)** | |  |  |
| Age at diagnosis^a^ | 27 (23, 34) | | - | 36 (32, 39) | | - | **0.014 ^c^** |
|  |  | |  |  | |  |  |
| Skin color |  |  |  |  |  |  | 0.747 ^d^ |
| Black | 13 | (81%) | 54%, 96% | 3 | (19%) | 4.0%, 46% |  |
| White | 11 | (85%) | 55%, 98% | 2 | (15%) | 1.9%, 45% |  |
| Missing | 5 | (71%) | 29%, 96% | 2 | (29%) | 3.7%, 71% |  |
|  |  |  |  |  |  |  |  |
| Gestational age |  | |  |  | |  | - |
| 1st trimester | 9 | (64%) | 35%, 87% | 5 | (36%) | 13%, 65% |  |
| 2nd trimester | 10 | (83%) | 52%, 98% | 2 | (17%) | 2.1%, 48% |  |
| 3rd trimester | 10 | (100%) | 69%, 100% | 0 | (0%) | 0.0%, 31% |  |
|  |  |  |  |  |  |  |  |
| Comorbidity |  |  |  |  |  |  | 0.076 ^d^ |
| No | 23 | (88%) | 70%, 98% | 3 | (12%) | 2.4%, 30% |  |
| Yes | 6 | (60%) | 26%, 88% | 4 | (40%) | 12%, 74% |  |
|  |  |  |  |  |  |  |  |
| Clinical form ^e^ |  |  |  |  |  |  | - |
| Localized | 24 | (77%) | 59%, 90% | 7 | (23%) | 9.6%, 41% |  |
| Disseminated | 5 | (100%) | 48%, 100% | 0 | (0%) | 0.0%, 52% |  |
|  |  |  |  |  |  |  |  |
| Antifungal use ^f^ |  |  |  |  |  |  | 0.084 ^d^ |
| No | 20 | (91%) | 71%, 99% | 2 | (9.1%) | 1.1%, 29% |  |
| Yes | 9 | (64%) | 35%, 87% | 5 | (36%) | 13%, 65% |  |

a. Median (Q1, Q3). b. CI = Confidence interval. c. Mann-Whitney test. d. Fisher’s exact test. e. Localized: fixed cutaneous and lymphocutaneous forms. Disseminated: disseminated cutaneous and extracutaneous/disseminated form. f. Women exposed to sporotrichosis medications contraindicated for pregnant women (itraconazole and saturated solution potassium iodide). p-value < 0.05 was considered significant. For variables with any value = 0, p-value was not used.
