# Supplementary material for: Sporotrichosis during pregnancy: A retrospective study of 58 cases in a reference center from 1998 to 2023
Source: PLoS Negl Trop Dis. 2024 Dec 20;18(12):e0012670. doi: 10.1371/journal.pntd.0012670 (PMC11661573; doi:10.1371/journal.pntd.0012670)
Supplement: S2 Table — (DOCX) [file pntd.0012670.s002.docx]

**S2 Table.** Analysis of demographic/clinical variables and neonatal outcomes of pregnant women with sporotrichosis treated at Fiocruz, INI, from 2001 to 2023.

| Variable | Newborn outcome | | 95% CI ^b^ | Newborn outcome | | 95% CI ^b^ | p-value |
| --- | --- | --- | --- | --- | --- | --- | --- |
|  | **Favorable**  **N = 28 (80%)** | |  | **Unfavorable**  **N = 7 (20%)** | |  |  |
| Age at diagnosis^a^ | 27.0 (23.5, 33.0) | | - | 33.0 (29.5, 37.0) | | - | 0.075 ^c^ |
|  |  | |  |  | |  |  |
| Skin color |  |  |  |  |  |  | 0.081 ^d^ |
| Black | 14 | (88%) | 62%, 98% | 2 | (13%) | 1.6%, 38% |  |
| White | 10 | (91%) | 59%, 100% | 1 | (9.1%) | 0.23%, 41% |  |
| Missing | 4 | (50%) | 16%, 84% | 4 | (50%) | 16%, 84% |  |
|  |  |  |  |  |  |  |  |
| Gestational age |  | |  |  | |  | - |
| 1st trimester | 8 | (73%) | 39%, 94% | 3 | (27%) | 6.0%, 61% |  |
| 2nd trimester | 10 | (71%) | 42%, 92% | 4 | (29%) | 8.4%, 58% |  |
| 3rd trimester | 10 | (100%) | 69%, 100% | 0 | (0%) | 0.0%, 31% |  |
|  |  |  |  |  |  |  |  |
| Comorbidity |  |  |  |  |  |  | 0.608 ^d^ |
| No | 23 | (82%) | 63%, 94% | 5 | (18%) | 6.1%, 37% |  |
| Yes | 5 | (71%) | 29%, 96% | 2 | (29%) | 3.7%, 71% |  |
|  |  |  |  |  |  |  |  |
| Clinical form ^e^ |  |  |  |  |  |  | - |
| Localized | 23 | (77%) | 58%, 90% | 7 | (23%) | 9.9%, 42% |  |
| Disseminated | 5 | (100%) | 48%, 100% | 0 | (0%) | 0.0%, 52% |  |
|  |  |  |  |  |  |  |  |
| Antifungal use ^f^ |  |  |  |  |  |  | 0.670 ^d^ |
| No | 19 | (83%) | 61%, 95% | 4 | (17%) | 5.0%, 39% |  |
| Yes | 9 | (75%) | 43%, 95% | 3 | (25%) | 5.5%, 57% |  |

a. Median (Q1, Q3). b. CI = Confidence interval. c. Mann-Whitney test. d. Fisher’s exact test. e. Localized: fixed cutaneous and lymphocutaneous forms. Disseminated: disseminated cutaneous and extracutaneous/disseminated form. f. Women exposed to sporotrichosis medications contraindicated for pregnant women (itraconazole and saturated solution potassium iodide). p-value < 0.05 was considered significant. For variables with any value = 0, p-value was not used.
